# Effectiveness of a screening protocol employed at a UK rescue centre to prevent introduction of strangles

**DOI:** 10.1111/evj.70080

**Published:** 2025-10-01

**Authors:** Luke A. McLinden, Jeremy G. Kemp‐Symonds, Janet M. Daly, Adam M. Blanchard, Andrew S. Waller, Sarah L. Freeman

**Affiliations:** ^1^ School of Veterinary Medicine and Science University of Nottingham Nottingham UK; ^2^ Veterinary Division Bransby Horses Lincoln UK; ^3^ Intervacc AB Stockholm Sweden

**Keywords:** guttural pouch, horse, screening, strangles, *Streptococcus equi*, *Streptococcus zooepidemicus*

## Abstract

**Background:**

Infection with *Streptococcus equi* subspecies *equi* (*S. equi*) is characterised by acute disease, with about 10% of infected animals remaining persistently infected. Clinically, infection with *S. equi* cannot readily be distinguished from infection caused by other respiratory pathogens, including *Streptococcus equi* subspecies *zooepidemicus* (*S. zooepidemicus*), equine influenza virus, and equine herpes virus. Screening protocols, with appropriate quarantining facilities, are important to detect horses infected with *S. equi* and avoid strangles outbreaks. Virulent strains of *S. zooepidemicus* can also cause strangles‐like presentations.

**Objectives:**

To evaluate the effectiveness of the screening process implemented at a UK welfare centre to prevent the introduction of strangles and strangles‐like presentations.

**Study Design:**

Retrospective cross‐sectional study.

**Methods:**

Clinical records of 626 equids admitted to a UK welfare centre between 2017 and 2021 and from horses that developed respiratory signs after admission were reviewed. The screening protocol, which included a clinical examination, paired serology samples (iELISA) taken 6 weeks apart, and bilateral guttural pouch endoscopy to identify abnormalities such as chondroids with lavage for qPCR and culture analysis for *S. equi* (and often *S. zooepidemicus*) was implemented during this time.

**Results:**

There were 34 screening‐positive equids. Of these, 24 (3.8%) were qPCR‐positive for *S. equi*, 8 were qPCR/culture positive for *S. zooepidemicus*, and 2 were qPCR/culture negative but had chondroids. Bilateral guttural pouch endoscopy, with qPCR analysis of lavage material, was an effective method of screening equids. There were no cases of strangles or strangles‐like presentations within the main herds after screening and admission of new horses.

**Main Limitations:**

Variation in the level of detail of clinical records.

**Conclusions:**

The screening process resulted in the identification of screening‐positive equids and maintained a strangles‐free herd. Further research is required to elucidate the significance of *S. zooepidemicus* infection in the guttural pouch.

## 1 INTRODUCTION

Equine strangles, caused by *Streptococcus equi* subspecies equi (*S. equi*), is a highly infectious disease that has significant implications for the health and welfare of equids across the globe.^1^, ^2^ The occurrence of strangles can be partly attributed to the presence of persistently infected animals, in which a viable population of *S. equi* persists and continues to shed, intermittently or continuously, following the apparent resolution of infection.^3^ The identification and treatment of persistently infected equids are important in the control of strangles and the prevention of outbreaks; this could be done in the aftermath of an outbreak or as a dedicated screening protocol.

There are many challenges associated with detecting animals persistently infected with *S. equi*, and there can be confusion around the effectiveness of the options available to caregivers and clinicians. Repeated nasopharyngeal lavage combined with qPCR and culture, on three occasions at least 2 weeks apart, has been shown to predict freedom from persistent *S. equi* infection,^4^ but does not reliably identify all equids that are persistently infected. Repeated nasopharyngeal lavage is necessary to mitigate against a high proportion of false negative results, but repeated testing is invasive and costly due to the need for recurrent veterinary action.^5^ The dual target iELISA for *S. equi* antigens A and C is unable to reliably identify persistently infected animals^6^ and its main use is in screening for exposure to *S. equi* following an outbreak to direct further testing. Guttural pouch endoscopy and lavage are considered the best methods for the detection of persistently infected horses, despite practical and economic implications, as they allow for a visual inspection of the guttural pouch alongside microbial analysis.^1^

In high‐risk settings, such as equine rescue centres, it may be appropriate to consider screening for *Streptococcus equi* subspecies *zooepidemicus* (*S. zooepidemicus*) alongside *S. equi*, as certain strains of *S. zooepidemicus* are particularly virulent and can cause strangles‐like presentations (e.g., guttural pouch infection, chondroid formation, lymph node abscessation).^7^

Bransby Horses UK is an equine welfare charity involved in the rescue, rehabilitation, and care of horses from around the UK. In 2008, Bransby Horses UK had a severe outbreak of strangles, with several cases of severe disease that led to the death of some affected horses. As a result, an isolation centre and screening programme was developed to evaluate and treat any horses screening‐positive for *S. equi* or *S. zooepidemicus* on admission and prior to entry onto the main facility (Text S1).

The aim of this study was to evaluate the effectiveness of a screening protocol at one of the UK's largest equine welfare centres, Bransby Horses UK. This protocol was developed and applied to new equids arriving at the facility with the aim of preventing the introduction of strangles and strangles‐like presentations into the main herds. The effectiveness of the screening protocol was measured by the absence of both detectable *S. equi/S. zooepidemicus* in released equids and clinical disease within the main herds over a 5‐year period.

## 2 MATERIALS AND METHODS

### 2.1 Study design

Using the STROBE checklist, this was a retrospective cross‐sectional study, reporting the isolation and screening protocol used to detect strangles and strangles‐like presentations in equids newly admitted to the rescue centre, the numbers of horses admitted, the results of screening tests, and, following the release of equids which were free from *S. equi*/*S. zooepidemicus*, the incidence of clinical signs of disease in the main herds population between 2017 and 2021.

### 2.2 Setting

The study was conducted in a UK equine rescue centre (Text S1), retrospectively collecting and analysing data from a 5‐year period (3 January 2017–14 December 2021).

### 2.3 Participants

The target population was equids that were screened for strangles on admission/re‐admission to the rescue centre. The inclusion criteria were equids that were subject to at least one aspect of this screening protocol (e.g., foals that were tested serologically but were too small to have endoscopy performed) were included. For the duration of the screening process (approximately 6 weeks), equids were housed in a dedicated quarantine unit on a rolling basis, alongside an onsite veterinary team. Once equids entered the main herds, all animals were checked at least once daily by a trained member of staff for signs of clinical disease. Any horses with clinical signs of respiratory disease (including nasal discharge, lymph node swelling, cough, change in demeanour) were reported to the on‐site veterinary team for investigation.

### 2.4 Variables

The screening protocol took place in a dedicated admission unit. The screening protocol included a clinical examination, paired blood samples taken at least 6 weeks apart for serology, endoscopic examination and lavage of both guttural pouches, with lavage fluid from each guttural pouch separately submitted to a diagnostic laboratory, typically for quantitative PCR (qPCR) analysis and culture for *S. equi* and *S. zooepidemicus* (Text S1). There were inconsistencies in what microbial testing was requested/performed, and not all samples were subjected to qPCR analysis and culture for both *S. equi* and *S. zooepidemicus*. Blood samples for serological testing by iELISA and guttural pouch lavage samples for microbial analysis were primarily sent to Rainbow Equine Hospital Limited (Spitfire House, Aviator Court, York, UK). Some samples were sent to other RCVS‐accredited equine hospitals, including Liphook Equine Hospital (Forest Mere, Liphook, Hampshire, UK) and Rossdales Laboratories (High Street, Newmarket, Suffolk, UK). Routine biochemistry and haematology samples were typically processed in‐house.

### 2.5 Diagnostic criteria

For the purpose of retrospective analysis of this screening protocol, equids were classified as screening positive or screening negative. Screening positive animals were equids that tested positive for *S. equi* or *S. zooepidemicus* through qPCR or culture, respectively, on guttural pouch lavage, or equids that had chondroids in their guttural pouch despite a negative test result. These criteria were deliberately broad as the aim of this protocol was to prevent the introduction of strangles or any strangles‐like presentations from entering the herd. Prior clinical histories were often limited or unavailable, particularly for equids rescued or transferred from unknown or unrecorded backgrounds, and therefore not used as part of carrier classification. Within the screening positive group, analysis was performed on equids that were positive on qPCR for *S. equi*.

Cases that were screening‐positive were kept in quarantine facilities and treated according to the protocol (Text S2). They were considered eligible for release into the main herds when guttural pouch qPCR and culture results for *S. equi*/*S. zooepidemicus* were negative. No animals were refractory to treatment during the study period.

To confirm the strangles‐free status of the herd, all cases of respiratory disease in the main herds between 2019 and 2021 were extracted and reviewed. This was done by using monthly key performance indicator (KPI) data provided by the rescue centre, and by evaluating the daily clinical diary between 2019 and 2021. Cases are logged in KPIs both by the internal welfare team and the veterinary team and cross‐referenced with any lab reports received. The data is logged as free‐text clinical notes, rather than a formal disease coding system. All entries were retrospectively reviewed by the authors, and only cases with clearly documented respiratory signs (e.g., nasal discharge, coughing, abnormal respiratory sounds) were classified as respiratory cases for the purpose of this analysis.

### 2.6 Data sources/measurement

Using the IDEXX Animana system, admitted equids were ranked by date of first admission. Each animal's clinical records could then be accessed to allow data retrieval. When clinical data on admitted equids could not be retrieved in this way, for example, if they had been readmitted or were deceased, clinical records were obtained from a paper copy.

A bespoke data capture form was created with numeric identification codes assigned to the admitted equids to provide anonymity and denote chronology. The form was separated into three sections: contextual, screening, and biochemistry and haematology data. Contextual data included information on key dates (admittance, sampling, and clinical examination). Age, breed, body condition score, sex, and neuter status of equids were also recorded in this section, where available. Screening data included results from the tests performed as part of the screening process (serology, endoscopy, and guttural pouch lavage), as well as a description of relevant clinical signs or guttural pouch abnormalities, such as lymphoid hyperplasia or the presence of chondroids. The results from qPCR analysis were designated either ‘positive’ or ‘negative’. Cycle threshold (CT) values were not available for many equids that were qPCR positive; therefore, these values were not captured. The optical density (OD) data from the serological test, a dual target iELISA for *S. equi* antigens A and C, were captured and later converted to a ‘positive’ (OD ≥ 0.5), ‘negative’ (OD < 0.3), or ‘equivocal’ (OD ≥ 0.3 < 0.5) result for analysis. Raw data from biochemistry and haematology tests, typically taken during the initial veterinary assessment and at the time of endoscopy, were captured and later converted to ‘elevated’, ‘normal’, or ‘lowered’, as defined by the laboratory's reference range for each parameter (Rainbow Equine Hospital, UK; Liphook Equine Hospital, UK; Rossdales Laboratories, UK).

### 2.7 Study size and bias

The data were collected from a single UK rescue centre. Many horses had limited details, including unknown ages or previous clinical histories. All data from horses seen during this period were retrieved and reviewed. Variations in protocols are reported. Some equids were not subject to all elements of the screening protocol, and clinical data were not recorded or retrievable in some cases (Table S1). The total number of equids included in each analysis (*N*) is presented.

### 2.8 Quantitative variables

Data were systematically cleaned by correcting transcription errors, standardising terminology, validating numerical entries, and excluding variables with missing values from relevant analyses. Continuous data were sorted into discrete categories to allow for descriptive and comparative analysis as part of an exploratory approach, and some categorical data, such as breed, were combined (Table S1a). Age was reported as categorical data, as accurate ages were unknown for many animals.

Two‐way pivot tables in Microsoft Excel v2211 were used to explore descriptive relationships and patterns between demographic/contextual variables (e.g., age, breed, body condition score) and screening outcomes (e.g., qPCR positivity).

### 2.9 Statistical analysis

The statistical software package GraphPad Prizm v9.4.1 was used to perform Fisher's exact, chi‐square, and chi‐square for trend tests to investigate the association between screening positive and host risk factors, biochemistry, and haematology results, and seropositivity, using a significance of *p* < 0.05. This analysis was also performed on animals that were *S. equi* qPCR positive on guttural pouch lavage.

## 3 RESULTS

### 3.1 Participants

This study evaluated the clinical records of 626 equids that had undergone at least one aspect of the screening protocol for *S. equi* and *S. zooepidemicus* and therefore met the inclusion criteria. A breakdown of the numbers of equids subjected to each part of the screening protocol is presented in Table S1b.

### 3.2 Descriptive data

The demographic data including age, breed, and gender is presented in Table S1.

## 4 MAIN RESULTS

### 4.1 Outcomes of the screening protocol

**FIGURE 1:**
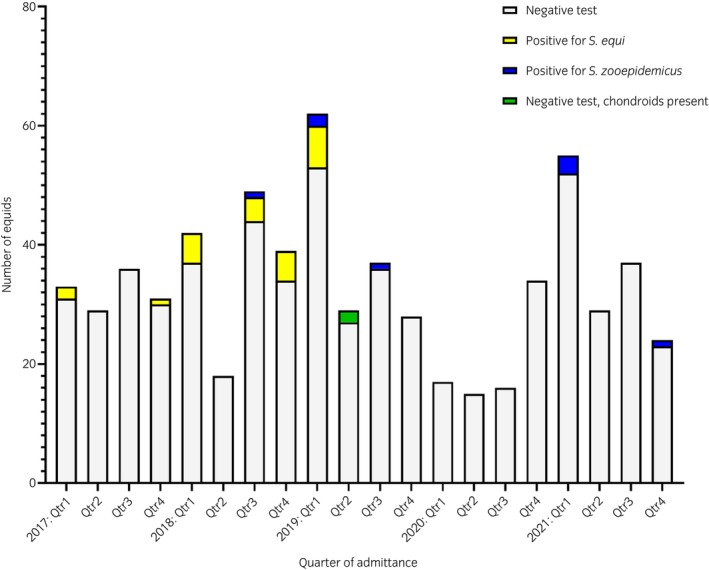
Results of guttural pouch lavage samples taken from 626 equids on admission to a UK rescue centre between 2017 and 2021 as part of a screening protocol employed to prevent the introduction of strangles and strangles‐like presentations.

**FIGURE 2:**
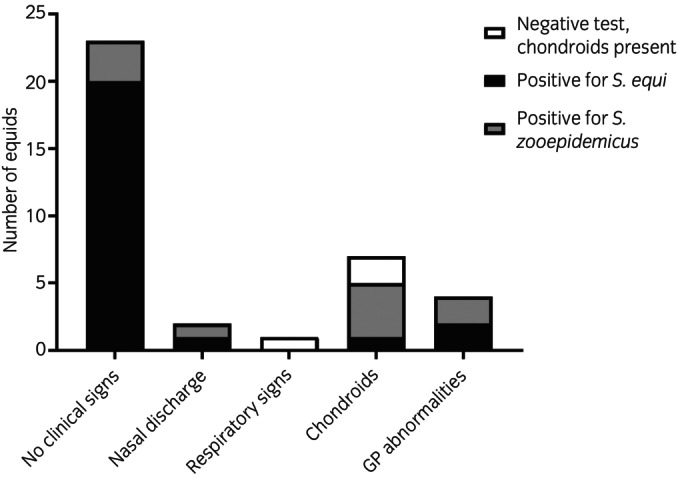
Clinical findings of equids that were classified as screening positive* on admission to a UK rescue centre between 2017 and 2021. *Screening positive animals were equids that tested positive for *S. equi* or *S. zooepidemicus* through qPCR or culture, respectively, on guttural pouch (GP) lavage, or equids that had chondroids in their GP despite a negative test result.

Thirty‐four of the 626 animals that met the screening criteria (5.4%) were classified as screening positive (Figure 1). Of these screening‐positive animals, 24 were *S. equi* qPCR positive, 8 were *S. zooepidemicus* qPCR or culture positive, and 2 had chondroids in their guttural pouches, but tested negative for *S. equi* and *S. zooepidemicus* by qPCR/culture of GPL samples. Thirty‐one of 34 (91%) screening‐positive animals displayed no signs of respiratory or systemic disease (Figure 2). Equids testing positive for *S. zooepidemicus* were significantly more likely to display clinical signs associated with bacterial respiratory infection (3/8) than those positive for *S. equi* (*p* = 0.02).

### 4.2 Risk factor analysis

Age, sex, breed, and body condition score were not significantly associated with horses that were screening‐positive or *S. equi* qPCR‐positive (Table 1), although lower body condition was more common among screening‐positive animals. No significant associations were found with any haematology or biochemistry parameters (Table S2).

**TABLE 1:** Demographic data for 626 equids that were screened for strangles on admission to a UK rescue centre between 2017 and 2021.

Category	Total	Screening positive^a^, *n* (%)	*S. equi* qPCR positive, *n* (%)
Age			
0–4	175	11 (6.3%)	8 (4.6%)
5–9	200	8 (4.0%)	6 (3.0%)
10–14	97	5 (5.2%)	5 (5.2%)
15–19	86	6 (7.0%)	3 (3.5%)
≥20	61	4 (6.6%)	2 (3.3%)
Total	619	34 (5.5%)	24 (3.9%)
Sex			
Female	293	18 (6.1%)	13 (4.4%)
Male entire	97	7 (7.2%)	5 (5.2%)
Male neutered	234	9 (3.8%)	6 (2.6%)
Total	624	34 (6.3%)	24 (3.8%)
Body condition score			
0.5, 1.0	38	3 (7.9%)	3 (7.9%)
1.5, 2.0	73	6 (8.2%)	3 (4.1%)
2.5, 3.0	204	13 (6.4%)	9 (4.4%)
3.5, 4.0	166	6 (3.6%)	5 (3.0%)
4.5, 5.0	90	3 (3.3%)	2 (2.2%)
Total	571	33 (5.8%)	22 (3.9%)
Breed type^b^			
Cob type	234	10 (4.3%)	8 (3.4%)
Large pony	110	9 (8.2%)	5 (4.5%)
Small pony	81	8 (9.9%)	6 (7.4%)
Light horse	41	1 (2.4%)	1 (2.4%)
Donkey type	19	3 (15.8%)	3 (15.8%)
Other	17	0	0
Sports horse type	14	2 (14.3%)	1 (7.1%)
Arab type	9	1 (11.1%)	0
Total	525	34 (6.5%)	24 (4.6%)

^a^ Screening positive animals were equids that tested positive for *S. equi* or *S. zooepidemicus* through qPCR or culture, respectively, on guttural pouch lavage, or equids that had chondroids in their guttural pouch despite a negative test result.

^b^ See Table S1a for categorisation of breed types.

### 4.3 Performance of *S. equi* dual‐target iELISA

There was no statistically significant association between screening‐positive animals and seropositivity of the *S. equi* dual‐target iELISA (*p* > 0.05, Fisher's exact test), whether a cut‐off of OD ≥0.5 or a less stringent cut‐off of OD ≥0.3 was used. There was also no statistically significant association between *S. equi* qPCR‐positive animals and seropositivity of the dual‐target iELISA (*p* > 0.05, Fisher's exact test). A greater proportion of *S. equi* qPCR‐positive animals were seropositive compared to the rest of the population on the first sample (13.6% vs. 7.3%), but this was not statistically significant and was not seen on the second sample (5.3% vs. 6.1%) (Table 2).

**TABLE 2:** ELISA serological results from 626 equids that were screened for strangles on admission to a UK rescue centre between 2017 and 2021.

Category of equid	Sample 1, *n*/*N* (%)	Sample 2, *n*/*N* (%)
*S. equi* qPCR positive	3/22 (13.6%)	1/19 (5.3%)
Screening positive	5/32 (15.6%)	1/28 (3.6%)
Screening negative	40/545 (7.3%)	30/494 (6.1%)

*Note*: Serological results for iELISA using *S. equi* antigens A and C for equids identified during the screening protocol as *S. equi* qPCR positive or as screening positive (*S. equi* qPCR positive o*r S. zooepidemicus* culture positive or having chondroids visible on endoscopy) compared with equids that were negative according to the screening protocol. Paired serum samples were taken at a minimum of 14 days apart.

Of the five animals identified through screening that were seropositive for *S. equi*, all were positive for antigen A, and only one also tested positive for antigen C. Among all 45 animals with a positive first sample, 28 were positive for antigen A only, nine were positive for both antigens, and eight were positive for antigen C only.

### 4.4 Effectiveness of the screening protocol

All animals which successfully completed the strangles‐screening protocol were released into the main herds. The effectiveness of the protocol was measured by the absence of clinical strangles within the main herds between 2019 and 2021. The retrospective review of clinical records (from monthly KPI records and the daily clinical diary records) between 2019 and 2021 identified 37 cases of respiratory disease within the rescue centre population (Figure S1). The rescue centre had a standardised respiratory disease protocol for identifying and investigating any horses with suspected respiratory disease. None of these were suspected to be strangles or strangles‐like cases by the on‐site veterinary team. In addition, there were no suspected or confirmed cases of strangles caused by *S. equi* or *S. zooepidemicus* between 2017 and 2019 (J. Kemp‐Symonds, personal communication, 2025), although respiratory disease was not formally recorded during this time.

## 5 DISCUSSION

### 5.1 Summary of findings

This study assessed the effectiveness of an admission screening protocol applied to 626 equids between 2017 and 2021 to prevent the introduction of strangles and strangles‐like presentations. Overall, 34 animals were identified as screening positive, with 24 testing positive on qPCR/culture for *S. equi*, 8 positive on qPCR/culture for *S. zooepidemicus*, and 8 having chondroids in their guttural pouch despite negative qPCR/culture results. There was no statistically significant association between screening positive equids and their age, breed, sex, body condition score, or with haematology or biochemistry abnormalities, or with serum *S. equi* specific ELISA results. This highlights the challenges in identifying horses with the potential to introduce *S. equi* and *S. zooepidemicus* infection, and the need for guttural pouch endoscopy sampling and diagnostic testing. This population had suffered severe strangles and strangles‐like outbreaks prior to the introduction of the screening protocol. There were no outbreaks of strangles or strangles‐like presentations within the main herds following screening, suggesting that the screening protocol was effective.

### 5.2 Limitations

There were a number of study limitations, which should be considered. Although horses were admitted from across the UK, the study was conducted in a single rescue centre; further studies reporting on protocols from across a range of centres and geographical locations are needed.

Due to the retrospective nature of the study involving data collected over a 5‐year period and the nature of the population, there was variation in the detail of data recorded, and some data were missing. Rescued horses frequently did not have previous histories or documentation available; therefore, any prior disease was often unknown, and age was often estimated.

There were also variations in the screening protocols. For example, young foals did not have guttural pouch endoscopy performed, and three different laboratories were used across the 5‐year period. All positive *S. equi* qPCR results originated from laboratories that were VETQAS‐accredited (http://apha.defra.gov.uk/apha-scientific/services/vetqas/index.htm), ensuring external quality assurance. CT values were not recorded alongside the reported *S. equi* qPCR results in clinical notes for many of the samples; therefore, the decision was made not to extract these data. The absence of CT values limited the assessment of bacterial load, and low bacterial load may have explained the lack of detection of *S. equi* by culture despite most samples being screened by both qPCR and culture. It has previously been reported that bacterial culture lacks sensitivity to detect *S. equi* infection.^8^, ^9^, ^10^, ^11^ Despite the long data collection period, it was confirmed that the *S. equi* qPCR and culture protocols used in the main laboratory to which samples were submitted did not change.

There was no active surveillance/sampling within the main herds, and therefore identification of strangles and strangles‐like presentations (or lack of) was dependent on routine health monitoring and investigation of any cases with possible clinical signs. To truly test the effectiveness of the screening protocol, an ideal study design would involve repeatedly resampling and testing all horses within the main herds. This was not possible due to the retrospective nature of the study, financial constraints, and ethical concerns associated with sampling clinically healthy animals in a welfare population. Some horses had prior handling and behavioural issues, and therefore all interventions were considered alongside the impact and benefit to the individual horse.

### 5.3 Defining strangles in a screening context

In this study, equids that went through the screening protocol were classified as screening positive or screening negative. Screening positive animals were equids that tested positive for *S. equi* or *S. zooepidemicus* through qPCR or culture, respectively, on guttural pouch lavage, or equids that had chondroids in their guttural pouch despite a negative test result. These criteria, developed by the rescue centre, were purposefully broad to minimise transmission into the main herds and identify any animals that were potential risks if introduced. To mitigate issues of over detection and misinterpretation, analyses were also performed with *S. equi* qPCR‐positive individuals only.

There is ongoing debate^12^, ^13^ over the clinical importance of *S. zooepidemicus* in relation to strangles. The decision to include animals that were *S. zooepidemicus*‐positive and test‐negative with chondroids within the screening positive category was based on a number of considerations. A positive result for *S. zooepidemicus* may reflect prior *S. equi* infection, with subsequent repopulation of the guttural pouch. Alternatively, there may be a mixed infection where *S. zooepidemicus* outcompetes *S. equi*, leading to false‐negative test results. In addition, *S. zooepidemicus* has been implicated in strangles‐like outbreaks, including cases with lymphadenopathy and nasal discharge.^7^ *S. equi* produces four superantigens: SeeH, SeeI, SeeL, and SeeM, which misdirect the equine immune response, enhancing the formation of lymph node abscesses.^14^, ^15^ Similarly, the formation of lymph node abscesses in the absence of *S. equi* was significantly associated with strains of *S. zooepidemicus* that produced the superantigens SzeN and SzeP.^7^ Furthermore, the presence of chondroids, even without confirmed microbial positivity for *S. equi*, poses a potential risk for harbouring residual infection. Given these factors, and the severity of a previous outbreak of strangles at the facility, the rescue centre preferred to take a comprehensive approach of applying follow‐up protocols to these strangles‐like cases and thus minimise the risk that potentially infectious animals were not identified and treated. This approach, while effective in minimising transmission risk, complicates subsequent interpretation and highlights the need for greater understanding of the clinical impact of these strangles‐like presentations.

In this study, screening‐positive animals were considered to be at risk of harbouring persistent *S. equi* infection even if they did not display any signs of respiratory or systemic disease. Prior clinical histories were often limited or unavailable, particularly for equids rescued or transferred from unknown or unrecorded backgrounds, and therefore not used as part of classifying animals as persistently infected.

### 5.4 Evaluation of screening protocol

The strangles and strangles‐like screening protocol employed at this UK rescue centre was effective in preventing clinical disease associated with *S. equi* and *S. zooepidemicus* within the resident herd over the 5‐year study period (2017–2021). No suspected or confirmed cases of strangles or strangles‐like presentations were identified, based on a review of clinical diary entries and KPI records between 2019 and 2021. Throughout the study period, all animals were closely monitored by trained carers and veterinary professionals. Any signs of respiratory disease were recorded and investigated between 2019 and 2021.

This screening protocol was introduced following a devastating outbreak of strangles at the centre around 20 years ago, which resulted in multiple fatalities. Since its implementation, no strangles outbreaks have occurred in the main herds, including during the study period and up to the time of article submission (J. Kemp‐Symonds, personal communication, 2025). It could be argued that shedding by the screening‐positive animals did not represent a sufficient ‘challenge dose’. However, strangles cases continued to occur at the same rate in the UK during the study period, with 867 strangles diagnoses compared to 807 in the preceding 3‐year period (2016–2018) reported by the Surveillance of Equine Strangles Network (https://app.jshiny.com/jdata/ses/sesview/), suggesting there was no change in the risk to the general equine population. The main herds consist of sentinel animals, the vast majority of which will, by now, be entirely naive immunologically to strangles. Many thousands of animals have now passed through the herds since the outbreak 20 years ago. The sustained absence of strangles and strangles‐like clinical cases across a large, vulnerable population suggests that the screening protocol has played an important role in minimising disease transmission.

While the absence of clinical disease is encouraging, it does not confirm freedom from *S. equi* or *S. zooepidemicus* infection. Microbial or serological surveillance of the resident herd would have been required to demonstrate complete absence of transmission, but this was not feasible in this population. Given the susceptibility of the cohort, however, the likelihood of undetected circulation of *S. equi* or virulent strains of *S. zooepidemicus* without any observable clinical signs is considered low.

### 5.5 Limitations of dual‐target *S. equi* iELISA

Serological testing has an important role in the management of strangles, particularly following outbreaks to identify recent exposure to *S. equi*.^1^, ^16^ However, the dual‐target iELISA is not reliable for detecting horses with persistent infection^6^, ^17^ and this study found no significant association between seropositivity and either *S. equi* qPCR positive or screening positive status.

The predominance of persistently infected animals (91%, 31/34) in the screening‐positive group may have contributed to the performance of the iELISA. It may be that some carriers have such a low bacterial load, or a dormant type of infection, that insufficient bacteria are shed to stimulate an immune response detectable by this assay. CT values were not available due to the retrospective nature of the study, so little can be inferred about the bacterial load in the equids that were screening positive.

Of the two targets in the *S. equi* iELISA, antigen A was responsible for detecting more screening‐ positive animals. Antigen C is encoded by part of the *SeM* gene region, which may undergo genomic decay in persistent infections.^18^ Antigen A is part of a sortase‐processed surface protein encoded by a segment of the *SEQ_2190* gene and may also be lost via a *SEQ_2180/SEQ_2190* recombination event.^18^ Due to the retrospective nature of the study, isolates were not collected for sequencing to investigate whether the decay of these targets contributed to the occurrence of false‐negative serology results.

### 5.6 Clinical significance of *S. zooepidemicus*

Eight screening‐positive equids tested positive for *S. zooepidemicus*. Samples obtained for the screening programme were not routinely subjected to qPCR detection of *S. zooepidemicus*; hence, most *S. zooepidemicus* infections were detected by culture. *Streptococcus zooepidemicus* is a very diverse subspecies with the potential to cause disease across multiple species and body systems.^12^, ^13^, ^19^, ^20^, ^21^, ^22^ In addition, *S. zooepidemicus* is commonly isolated from the respiratory mucosa of healthy and diseased equids alike^23^, ^24^; consequently, debate exists over whether it is a commensal or a primary or opportunistic pathogen.^12^ Strains of *S. zooepidemicus* have been shown to cause outbreaks of disease across the globe, with notable examples in Iceland,^25^ Sweden,^13^ the UK,^13^ and Ethiopia.^26^ In this study, *S. zooepidemicus*‐positive equids were more likely to have clinical signs than those that were *S. equi* positive.

As implemented, the screening protocol appears to have enabled animals infected with *S. zooepidemicus* to be intercepted, potentially also preventing outbreaks caused by this pathogen. Questions remain over whether *S. zooepidemicus* can establish persistent infection of the guttural pouches as a primary pathogen or whether the presence of this organism is secondary to *S. equi*, repopulating the guttural pouch as the host immune response exerts selective pressure on *S. equi*. Further research is required; for example, obtaining multiple genome sequences of *S. zooepidemicus* isolated from chondroids to determine if certain strains are more able to persist within chondroids and whether the production of certain virulence factors, such as SzeN and SzeP, is more frequently encoded by isolates of *S. zooepidemicus* that are recovered from the guttural pouches.

### 5.7 Host factor analysis

This study did not identify age, breed, sex, or body condition score as risk factors for animals that were *S. equi* qPCR or screening positive. Age has been suggested as a risk factor for acute strangles infection,^27^ but the association is inconsistent.^28^ Strangles has been linked to low body condition score in an observational study.^27^ A more recent study^28^ suggested that equids with a higher body condition score were more likely to be in work and mixing with other equids and thus more likely to be exposed to *S. equi*, but this was in a developing country setting (Lesotho). The lack of significant association between age, sex, breed, or body condition score and *S. equi* carriage in this study highlights the importance of effective screening protocols for all equids, regardless of host factors.

This study also did not find a significant association between any biochemistry or haematology result and animals that were *S. equi* qPCR or screening positive. Haematological parameters such as hyperfibrinogenaemia and neutrophilia have been previously linked to acute strangles infection.^5^, ^29^ Our study population consisted of a substantial proportion of carrier animals, and the lack of significant changes in markers of inflammation or infection supports previous findings.^17^

## 6 CONCLUSION

This retrospective study describes a strangles and strangles‐like screening protocol applied to equids arriving at a UK rescue centre. The protocol identified 5.4% of horses as screening‐positive and considered at risk of introducing strangles or strangles‐like presentations into the main herd. Most cases that were screening positive had no external clinical signs of respiratory disease, and screening positive status was not associated with signalment, haematology, biochemistry or serum ELISA results. Since the introduction of the screening protocol, there have been no clinical outbreaks of strangles or strangles‐like presentations within the main herd. The findings highlight the importance of guttural pouch endoscopy, sampling and testing towards minimising the risk of *S. equi* introduction into susceptible populations. Through the adoption of screening protocols, as well as other long‐term control measures, such as the vaccination of unexposed animals, the prevention and control of strangles is increasingly achievable. Further work is needed to determine the significance of *S. zooepidemicus* infections of the guttural pouch and whether this subspecies is acting as a primary pathogen or secondary to *S. equi*.

## FUNDING INFORMATION

This study is funded by the University of Nottingham as part of an MRes Scholarship.

## CONFLICT OF INTEREST STATEMENT

The authors declare no conflicts of interest.

## AUTHOR CONTRIBUTIONS

**Luke A. McLinden:** Conceptualization; methodology; data curation; formal analysis; visualization; writing – original draft; writing – review and editing. **Jeremy G. Kemp‐Symonds:** Conceptualization; methodology; formal analysis; supervision; visualization; writing – review and editing. **Janet M. Daly:** Conceptualization; methodology; formal analysis; supervision; funding acquisition; visualization; writing – review and editing. **Adam M. Blanchard:** Conceptualization; methodology; supervision; writing – review and editing. **Andrew S. Waller:** Conceptualization; methodology; supervision; writing – review and editing. **Sarah L. Freeman:** Conceptualization; methodology; formal analysis; supervision; funding acquisition; visualization; writing – review and editing.

## DATA INTEGRITY STATEMENT

Luke McLinden had full access to all the data in the study and takes responsibility for the integrity of the data and the accuracy of the data analysis.

## ETHICAL ANIMAL RESEARCH

The study protocol was approved by the School of Veterinary Medicine and Science Ethics Committee.

## INFORMED CONSENT

Consent provided by representatives of the welfare centre caring for these horses.

## Supporting information


**Figure S1.** Number of equids presenting with signs of respiratory disease within a UK rescue centre between 2019 and 2021.

**Table S1.** Categorisation of breeds and demographic data for 626 equids admitted to a UK rescue centre between 2017 and 2021.

**Table S2.** Biochemistry and haematology results for equids screening positive on admission to a UK rescue centre between 2017 and 2021.

**Text S1.** Effectiveness of a screening protocol employed at a UK rescue centre to prevent introduction of strangles.

**Text S2.** Treatment protocol from Bransby Horses UK ‘Equine Strangles Procedure’ documentation.

## Data Availability

The data that support the findings of this study are openly available in Nottingham Research Data Management Repository at https://rdmc.nottingham.ac.uk/handle/internal/12078.
