# Supplementary material for: Effectiveness of a screening protocol employed at a UK rescue centre to prevent introduction of strangles
Source: Equine Vet J. 2025 Oct 1;58(2):466–75. doi: 10.1111/evj.70080 (PMC12892369; doi:10.1111/evj.70080)
Supplement: Supplementary file 1 — Figure S1. Number of equids presenting with signs of respiratory disease within a UK rescue centre between 2019 and 2021. [file EVJ-58-466-s004.pdf]

Equine Veterinary Journal
BEVA

Figure S1: Number of equids presenting with signs of respiratory disease within a UK rescue centre between 2019 and 2021.

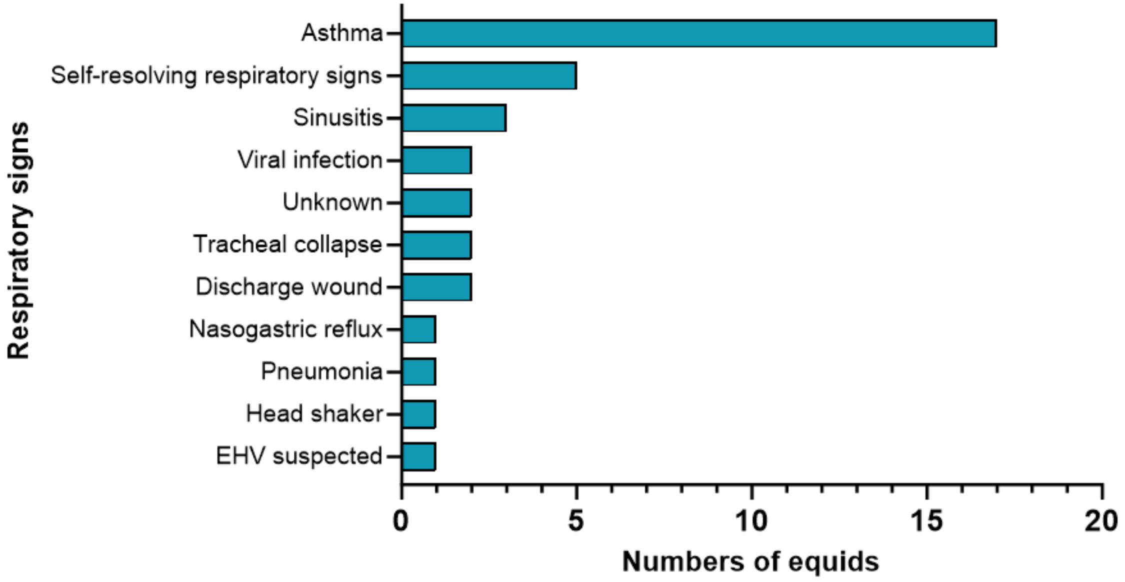

EHV, Equine Herpes Virus 1 or 4.
