# Supplementary material for: Effectiveness of a screening protocol employed at a UK rescue centre to prevent introduction of strangles
Source: Equine Vet J. 2025 Oct 1;58(2):466–75. doi: 10.1111/evj.70080 (PMC12892369; doi:10.1111/evj.70080)
Supplement: Supplementary file 2 — Table S1. Categorisation of breeds and demographic data for 626 equids admitted to a UK rescue centre between 2017 and 2021. [file EVJ-58-466-s002.pdf]

Equine Veterinary Journal BEVA

Table S1: Categorisation of breeds and demographic data for 626 equids admitted to a UK rescue centre between 2017 and 2021.

Table S1a: Categorisation of breeds/types of equids as described in the clinical records of 626 equids admitted to a UK rescue centre between 2017 and 2021.

Classification	Breed or Type
Donkey type	Donkey, Mule
Small pony	Shetland (X), Welsh Section A, Miniature horse, Miniature Shetland, Falabella
Large pony	Pony, Welsh Section B, C, Welsh (X), Fell, Connemara (X), Riding pony, Highland (X), New Forest (X), Dartmoor, Dales, Haflinger
Arab type	Arab (X), Anglo Arab
Cob type	Cob (X), Welsh Section D, Welsh Cob
Light horse	Thoroughbred (X), Morgan, Appaloosa, Hackney (X), Trotter, Standardbred, Riding Hack
Sports horse type	Warmblood (X), Irish Sport Horse, Andalusian, Lipizzaner, Oldenburg, French, Friesian, Irish Draught (X), Irish (X), Westphalian, Selle Française, Holstein, Hanoverian (X), Gelderlander, Cleveland Bay (X), Trakehner
Other	Shire (X), Clydesdale (X), Percheron (X), unspecified

'X' = crossed with another breed/type

Table S1b: Population demographics and clinical data retrieved from a population of 626 equids admitted to a UK rescue centre.

Variable	Recorded	Not recorded/ unable to retrieve data
Age	619	7
Breed	617	9
Sex	624	2
Body condition score	571	55
Endoscopy and guttural pouch lavage	561	65
Serology at initial vetting in	622	4
Serology at time of endoscopy (~ 6 weeks after admission)	531	95
Biochemistry and haematology	360	266
