# Supplementary material for: Effectiveness of a screening protocol employed at a UK rescue centre to prevent introduction of strangles
Source: Equine Vet J. 2025 Oct 1;58(2):466–75. doi: 10.1111/evj.70080 (PMC12892369; doi:10.1111/evj.70080)
Supplement: Supplementary file 3 — Table S2. Biochemistry and haematology results for equids screening positive on admission to a UK rescue centre between 2017 and 2021. [file EVJ-58-466-s005.pdf]

Equine Veterinary Journal BEVA

Table S2: Biochemistry and haematology results for equids screening positive on admission to a UK rescue centre between 2017 and 2021.

Table S2a: Abnormal biochemistry results and their association with screening positive in a population of 626 rescue equids admitted between 2017 and 2021.

**Parameter**	**Number (%)**	
	**Screening positive* (N = 20)**	**Screening negative (N = 340)**
Total protein	6 (30%)	76 (22.4%)
Albumin	4 (20%)	69 (20.3%)
Globulin	6 (30%)	107 (31.5%)
Fibrinogen	9 (45%)	163 (47.9%)
Serum amyloid A	4 (20%)	47 (13.8%)
Aspartate transaminase	13 (65%)	175 (51.5%)
Gamma-glutamyltransferase	9 (45%)	130 (38.2%)
Creatine kinase	5 (25%)	65 (19.1%)
Glutamate dehydrogenase	4 (20%)	105 (30.9%)
Alkaline phosphatase	2 (10%)	44 (12.9%)

*Screening positive animals were equids that tested positive for *S. equi* or *S. zooepidemicus* through qPCR or culture, respectively, on guttural pouch lavage, or equids that had chondroids in their guttural pouch despite a negative test result.

Table S2b: Abnormal haematology results and their association with screening positive* in a population of 626 rescue equids admitted between 2017 and 2021.

**Parameter**	**Number (%)**	
	**Screening positive* (N = 20)**	**Screening negative (N = 340)**
Red blood cells	1 (5%)	25 (7.4%)
Packed cell volume	3 (15%)	67 (19.7%)
Haemoglobin	3 (15%)	85 (25%)
Red blood cell distribution width	13 (65%)	207 (60.9%)
Platelets	2 (10%)	13 (3.8%)
White blood cells	6 (30%)	109 (32.1%)
Neutrophils	2 (10%)	56 (16.5%)
Monocytes	4 (20%)	49 (14.4%)

*Screening positive animals were equids that tested positive for *S. equi* or *S. zooepidemicus* through qPCR or culture, respectively, on guttural pouch lavage, or equids that had chondroids in their guttural pouch despite a negative test result.
