# Supplementary material for: Effectiveness of a screening protocol employed at a UK rescue centre to prevent introduction of strangles
Source: Equine Vet J. 2025 Oct 1;58(2):466–75. doi: 10.1111/evj.70080 (PMC12892369; doi:10.1111/evj.70080)
Supplement: Supplementary file 4 — Text S1. Effectiveness of a screening protocol employed at a UK rescue centre to prevent introduction of strangles. [file EVJ-58-466-s001.pdf]

Equine Veterinary Journal BEVA

Text S1: Effectiveness of a screening protocol employed at a UK rescue centre to prevent introduction of strangles.

1. Bransby Horses UK

Bransby Horses UK is one of the UK's largest equine welfare charities comprising a primary centre with over 300 equids and a subsidiary site with around 50 equids. In addition, around 500 horses are fostered out at any one time.

2. Strangles screening protocol

Bransby Horses UK established a strangles screening protocol following a severe strangles outbreak in 2008 that resulted in significant morbidity and mortality. The charity has a dedicated admission unit which all horses pass through upon arrival for veterinary assessment and strangles testing. Once equids enter the general herd, all animals are checked at least once daily by a trained member of staff.

The screening protocol included a clinical examination, paired serology samples (iELISA) taken six weeks apart, and endoscopically guided guttural pouch lavage of both guttural pouches which were sent for quantitative PCR and culture analysis. For the duration of the strangles screening process (approximately six weeks), equids were housed in a dedicated quarantine unit, under the care of an onsite veterinary team.

Equids that were subject to at least one aspect of this screening protocol were included in this study, for example foals that were tested serologically but were too small to have endoscopy performed. Equids that were admitted but not tested for strangles, such as those that were euthanised shortly after arrival, were not included in this study. Equids that were admitted more than once during this timeframe, for example animals that had been to hospital or fostered out and returned, were treated as a separate case for each time they went through the screening process.
