# Supplementary material for: Effectiveness of a screening protocol employed at a UK rescue centre to prevent introduction of strangles
Source: Equine Vet J. 2025 Oct 1;58(2):466–75. doi: 10.1111/evj.70080 (PMC12892369; doi:10.1111/evj.70080)
Supplement: Supplementary file 5 — Text S2. Treatment protocol from Bransby Horses UK ‘Equine Strangles Procedure’ documentation. [file EVJ-58-466-s003.pdf]

Text S2: Treatment protocol from Bransby's 'Equine Strangles Procedure' documentation.

Developed by the Bransby veterinary team, shared with all staff involved in care and welfare of Bransby horses. Reviewed annually.

2.5 Treatment of S. equi infection and guttural pouch empyema

The mainstay of treatment of equines with guttural pouch empyema and clinical strangles is nursing and supportive care. Provision should be made for appropriate analgesia and management of the fever. Adjustments to management should be made based on the severity of clinical signs, for example raising feed buckets if neck range of motion is compromised or providing additional soft feeds for equines with pharyngitis or dysphagia. These changes should be implemented on the advice of the treating veterinary surgeon.

Systemic antibiotic treatment should not be used to treat confirmed cases of S. equi as this has the potential to drive carrier status in preference to disease clearance. The only exception would be if the equine developed severe compromising primary disease e.g. lymph node abscesses impacting on breathing/swallowing or secondary disease e.g. pneumonia, in which case antibiotic treatment should be instigated on the recommendation of the treating veterinary surgeon, ideally in line with culture and sensitivity results.

The first line treatment for management of guttural pouch empyema should be high volume lavage of the guttural pouches, either endoscopically or via previously placed foley catheters if there is excessive purulent material/obvious ruptured lymph nodes in the guttural pouches that the treating veterinary surgeon feels would benefit from an extended course of flushing. Warm water should be used, with no additional disinfectants or antibiotics added. A garden pump sprayer can be attached to the endoscope channel or the foley catheter and used to provide high volume lavage.

Liquid antibiotics e.g. depocillin should never be instilled into the guttural pouches due to the risk of colitis from gastrointestinal tract exposure. A gel based penicillin formulation can be used in the guttural pouches, but this should be reserved for clinical cases refractory to high volume lavage or carrier status equines that are refractory to high volume lavage.

Should the equine develop severe disease leading to respiratory distress or systemic compromise, there may be a requirement for hospitalisation for intensive care, in which case the Referrals procedure should be followed. The referral hospital should be notified that this is a case of infectious disease so that they can follow their own isolation protocol on receipt of the affected equine.

Affected equines should remain under strict isolation and barrier nursing conditions until they have been confirmed clear of the infection. Repeat guttural pouch endoscopy and sampling should be performed a minimum of 3 weeks after the resolution of nasal discharge. If the results returned are negative, the equine can then leave barrier nursing. If the results returned are positive on either PCR or culture, please see 2.6.

If foals are affected with clinical signs of S. equi and are too small to undergo guttural pouch endoscopy and flushing, an alternative treatment method must be considered. In these cases systemic antibiotic treatment may need to be considered if the foal is systemically sick or compromised, in addition to non-steroidal anti-inflammatory therapy. Given the propensity for sick foals to deteriorate rapidly, consideration should be given to referral for intensive care if the treating veterinary surgeon has concerns about ongoing treatment and management at Bransby Horses. Diagnosis can be confirmed via PCR and culture of nasal discharge, nasopharyngeal swabbing or of abscess discharge, alongside a rising titre on serology, although all foals showing these clinical signs should be considered positive for S. equi even without a confirmed diagnosis. Foals with a confirmed diagnosis of S. equi or those having demonstrated likely clinical signs should then remain at the Arc

with their in contact group until it is possible to perform guttural pouch endoscopy to confirm the foal has cleared the infection and has not retained carrier status or chondroids. If at this time a foal is found to have chondroids or be a carrier then the treatment protocol is the same as for adult equines.

2.6 Carrier status and chondroids

Some equines previously exposed to S. equi infection will not clear the infection and will go on to become carriers or to develop chondroids. Carrier status horses retain the bacterium within the guttural pouches without showing clinical signs of disease and are capable of shedding the bacterium and infecting other equines. Chondroids are balls of insipated pus harbouring the bacterium, which can form after clinical disease if the purulent material is not flushed from the pouches and cannot then be removed from the pouches without manual excavation.

If chondroids are identified on guttural pouch endoscopy, every effort should be made to remove them at this time transendoscopically. There may be a requirement to do this in a staged procedure if there are multiple chondroids or if both pouches are affected to reduce duration of sedation each time and minimise guttural pouch trauma. If there are large numbers of chondroids or they are too large to be removed transendoscopically the equine may need to be referred for surgical removal. If this is the case then the Referrals Procedure should be followed. If chondroid material can be retrieved, then this should be macerated and placed in a bacterial transport media and submitted alongside pooled washes of the guttural pouches for PCR and culture. Once the chondroids have been removed, high volume lavage of the guttural pouches should be performed as per 2.4. if these samples return a positive result, repeat sampling of the guttural pouches should be carried out 10-14 days later. If these samples return a negative result, this duration can be shortened and if a second negative result is returned then the equine can leave the ARC.

If an equine is identified as a carrier without chondroids or guttural pouch empyema (a positive PCR or culture result on pooled guttural pouch washes) then they will require further treatment before leaving the ARC. These equines should undergo high volume lavage via endoscopy before repeat sampling 10-14 days later.

Antibiotics should not be instilled into the guttural pouches unless the equine is considered refractory to high volume lavage with a positive result obtained on the second sampling. In these cases, repeat high volume lavage should be performed with the addition of gel based penicillin treatment and a 10 day course of systemic antimicrobials before repeating sampling at 14 days. A liquid antibiotic should never be instilled into the guttural pouches due to the risk of colitis.

Vaccination can be considered with Strangvac in chondroid or carrier cases (see 2.2. Vaccination).

2.7 Streptococcus equi var zooepidemicus

S. zooepidemicus is a bacterium in the same family as S. equi that has the potential to cause respiratory infection in equines, although one of less significance than S. equi. If an equine with clinical signs of respiratory disease tests positive for S. zooepidemicus, they should continue being barrier nursed until the resolution of clinical signs and a negative test result (guttural pouch endoscopy or nasopharyngeal swab depending on the initial diagnostic test taken). The treatment regimen should be at the discretion of the treating veterinary surgeon based on the severity and location of disease. Where possible antibiotic treatment should be avoided in the first instance unless respiratory disease is severe, and if it is indicated it should be done in line with the BEVA Protect Me guidelines and culture and sensitivity results to guide antibiotic choice.

In some cases, equines may return a culture result for S. zooepidmicus on guttural pouch sampling. If this is the case, the same protocol should be followed as for a positive S. equi result with high volume lavage and repeat sampling 10-14 days later. Equines with a positive S. zooepidemicus result should not leave the ARC until a negative result has been returned. Consideration can be given to treating these cases with Strangvac
